# Two-domain mechanics of a spherical, single chamber heart with applications to specific cardiac pathologies

**DOI:** 10.1186/2193-1801-2-187

**Published:** 2013-04-26

**Authors:** Steffan Puwal

**Affiliations:** Department of Physics, Oakland University, Rochester, MI 48309 USA

## Abstract

Continuum approximations of tissue consider responses averaged over many cells in a region. This simplified approach allows consideration of macroscopic effects, such as deformation or action potential propagation. A bidomain (sometimes known as biphasic) approach retains the macroscopic character of a continuum approximation while allowing one to consider microscopic effects; novel behavior arising from interactions between the intracellular and extracellular spaces can also be noted. I consider a spherical, single chamber heart with the new mechanical bidomain model in four separate pathologies: hypertension, hypovolemic hypotension, and hypertrophic and dilational cardiomyopathies. Analytic solutions of intracellular and extracellular displacements and hydrostatic pressures are presented; the distributions describe elastic deformation and hydrostatic fluid pressure buildup of the extracellular collagen matrix and the intracellular muscle under simplified spherical geometry. Potential applications, such as stretch activated membrane channels, are also noted.

## Introduction

Continuum models of tissue average the behavior of interest over many cells. Typically this limits consideration to macroscopic effects. A bidomain approach to cardiac muscle is necessary to explain behavior that results from unequal intracellular and extracellular spaces (Tryanova et al. 1993). For example, proarrhythmic responses to external stimuli have been predicted with the electrical bidomain model when the intracellular and extracellular spaces have unequal anisotropy ratios for conductivity (Tryanova et al. 1993). At the sub-cellular and molecular levels of organization discrete models are necessary to describe the response of cardiac muscle. For example, Eshelby (Eshelby 1957) presented an early model useful for considering the elasticity of inclusion bodies (which, here, would be individual cells). Others (Ohayon & Chadwick 1988; Chadwick 1982; Chadwick 1981; Panfilov et al. 2005; Panfilov et al. 2007; Nash & Panfilov 2004; Bini et al. 2005; Glass et al. 1991; Guccione et al. 1991; Arts et al. 1979; Latimer et al. 2003) have presented continuum elastic models which are monodomain in the sense that, not only do they average over many cells, but they also average together both the intracellular and extracellular spaces. These models allow one to consider anisotropy, though they typically assume a strain linearly proportional to the applied stress. A background isotropic term is usually associated with the extracellular collagen mesh, while anisotropic terms represent the muscle fiber. Under sufficient stress, most elastic materials will behave nonlinearly, or even plastically deform. However, linear models such as these are useful for determining first order responses because we can find an analytic solution. Nonlinear models are typically limited to numerical results.

Puwal and Roth (Puwal & Roth 2010; Punal & Roth 2012; Puwal & Roth 2013) have previously presented a linear mechanical bidomain model that separately considers the intracellular and extracellular spaces as elastic continua coupled by a spring force whose magnitude is proportional to the displacement difference of the two spaces. (Note that the terms “monodomain” and “bidomain” are standard terms in the cardiac research literature. Those familiar with elasticity theory may recognize these terms as “monophasic” and “biphasic”.) The advantage of the bidomain approach is that we may consider a particular microscopic parameter averaged over many cells, while still discussing macroscopic effects. In this case, the microscopic parameter is the nature of the force coupling elastic deformations of the intracellular and extracellular spaces.

This new model may provide insight into how the diseased state may translate into large membrane stresses, thereby leading to ectopic electrical activity. Here, I consider a single chamber, spherical heart with a wall of finite thickness. Of course, the human heart is four chambered and not spherical. However, an analytic solution of elastic deformation is not likely to be found by considering the true geometry of the heart and a spherical model will retain the fundamental characteristics of the heart (e.g. increasing chamber pressure leads to an outward displacement of the muscle). The analytic solution presented here is intended only to approximate the response of the human heart. The displacement fields and hydrostatic pressures are found to be in general agreement with what we expect—namely, increasing active tension causes the heart to contract and increasing chamber pressure causes the heart to expand. The analytic solution allows us to determine the pressure and displacement fields and qualitatively comment on hearts with underlying pathologies. I conclude with a discussion of how these solutions may be applied to four specific pathologies in particular: hypertension, hypovolemic hypotension, hypertrophic cardiomyopathy, and dilational cardiomyopathy. My discussion of these pathologies is limited by the fact that I do not consider remodeling of the elastic parameters during progression of the diseased state.

## Methods

The results presented here are derived equations based on theory and, therefore, require no ethical considerations.

A very general definition of a cell would describe an interior space for metabolic reactions to take place which is separated from its surrounding extracellular space by a membrane (Guyton & Hall 2006; Nelson & Cox 2008). In muscle cells (myocytes), the sarcolemma consists of the typical phospholipid bilayer present in most cell membranes as well as a polysaccharide coat with collagen proteins (Guyton & Hall 2006; Nelson & Cox 2008). Cardiomyocytes have the additional distinguishing feature of intercalated disks which connect the intracellular spaces of adjacent cardiomyocytes to form a single continuous intracellular space (Guyton & Hall 2006). Our model of elasticity will consider the differing elasticity of components of the intracellular and extracellular spaces and discuss how mechanical disturbances in one space are translated into another.

### The extracellular space

The extracellular space is that portion of the tissue outside of the cell membrane. The cardiomyocyte extracellular space contains fluid (including large amounts of water), dissolved ions, polysaccharides and collagen (Guyton & Hall 2006; Nelson & Cox 2008). Important for our considerations will be the collagen matrix. Collagen is a protein found in all connective tissue as well as the extracellular space of cardiomyocytes (Nelson & Cox 2008). While there are several types of collagen, the structure of collagen generally involves three *α*-chain proteins very tightly twisted around each other and forming a coiled structure. This tight coil provides great tensile strength. The *α*-chains within a collagen fibril and across adjacent fibrils are linked by covalent bonding. The linking between adjacent collagen fibrils arranged at a random relative orientation forms a tightly woven mesh of collagen exhibiting relatively isotropic elasticity when compared to the intracellular space (Nelson & Cox 2008).

The collagen mesh is essentially a polymer consisting of covalently bonded subunits of collagen fibers and the covalent bonding in polymers is often approximated with a linearly elastic model (Nelson & Cox 2008; Ibach & Luth 2009). Thus, if  is the extracellular stress tensor,  is the extracellular strain tensor and *G* is the shear elastic modulus we may write1

where  is the identity matrix and *q* is the extracellular hydrostatic pressure (Reismann & Pawlik 1980; Arya 1998; Arfken & Weber 2001). In general, collagen is quite resistant to elastic deformation and the shear modulus will be large—on the order of 10^4^ Pascals (Latimer et al. 2003; Puwal & Roth 2010).

### The intracellular space

The intracellular space of a cardiomyocyte is dominated by structures which facilitate the contraction of the muscle. Normal contraction is the result of actin and myosin protein filaments sliding past each other after an action potential triggers the release of calcium ions from the sarcoplasmic reticulum. The actin and myosin filaments are aligned in a side-by-side arrangement by an accessory protein called titin (Guyton & Hall 2006; Nelson & Cox 2008) (characterized as “very springy” (Guyton & Hall 2006)). These bundles of aligned contractile filaments (which may be visualized on an electron micrograph (Nelson & Cox 2008)) give the intracellular space a preferred direction of elasticity. We regard stresses which stretch the intracellular space as also stretching the longitudinally directed actin-myosin complexes and the covalently bonded titin proteins. While the intracellular space will exhibit some background isotropy, the relative displacements of two coupled isotropic spaces are not particularly interesting, so we will focus our attention on the anisotropic nature of the intracellular space.

**Figure 1:**
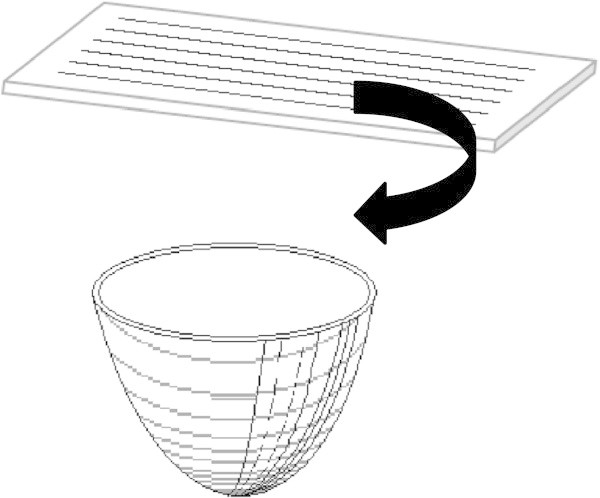
**Folding of a sheet of heart tissue to make a spherical heart.** An approximation of the orientation of muscle fibers in cardiac muscle may be obtained by considering a sheet of muscle wrapped to form a spherical chamber. In such an arrangement there is no single muscle fiber orientation. Rather, the fiber orientation changes with depth in the heart muscle. I assume fibers will point in all directions perpendicular to the chamber wall with depth and that this arrangement may be approximated as isotropic in the angular and azimuthal directions.

As we did with the extracellular space, we will consider the intracellular stress to be linearly proportional to the intracellular strain. The intracellular space will be modeled with extreme anisotropy derived under the assumption that the wall of the heart is formed from a folded sheet of cardiac tissue (Guyton & Hall 2006), as shown in Figure 1. Since the tissue is folded onto itself to create the heart chamber, the direction of the muscle fibers will change with depth into the muscle wall. We can simplify our model slightly by assuming a homogeneous, spherical heart wall with isotropic elastic behavior in the directions parallel to the muscle wall, and highly anisotropic behavior in the direction perpendicular to the muscle wall (Reismann & Pawlik 1980; Arya 1998; Arfken & Weber 2001). That is, purely radial stresses are assumed to be independent of strain. For a spherical chamber wall we can write

**Figure 2:**
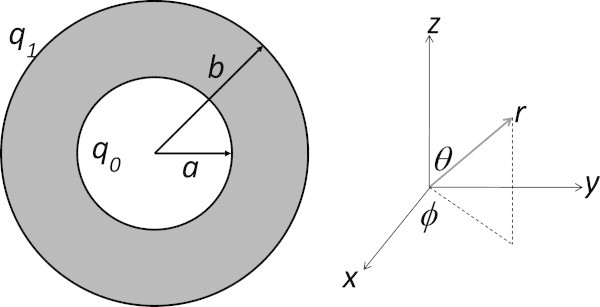
**The geometry of the spherical heart.** The spherical heart model will make use of spherical coordinates standard in physics, with the azimuthal angle off the *x*-axis. The radial symmetry of the problem presents a chamber of radius *a* maintained at a fluid pressure *q*_0_, a muscle thickness (*b* – *a*) and an outer enclosing pericardial sac maintained at fluid pressure *q*_1_.

234

where (*r, θ*) are the usual spherical coordinates shown in Figure 2, *p* is the intracellular hydrostatic pressure, *T*_0_ is the active tension for the muscle fibers, *μ* is the elastic modulus of the chamber wall in the diretion parallel to the wall itself (a term that is essentially a hybrid of the Young’s modulus and the shear modulus),  is the intracellular stress tensor, and  is the intracellular strain tensor. The symmetry of the problem allows us to assert that the intracellular and extracellular displacement fields have no azimuthal or angular component and that the displacements are a function of the radial coordinate *r* only (Reismann & Pawlik 1980).

56

The intracellular and extracellular strains may be written789101112

where *∂*_*r*_ is the partial derivative with respect to the radial coordinate (Reismann & Pawlik 1980). And, therefore, the off-diagonal terms of the stress tensors are found to be (Reismann & Pawlik 1980).1314

### Coupling between the intracellular and extracellular spaces and the equations of mechanical equilibrium

The intracellular and extracellular spaces of cardiac muscle are not mechanically isolated from each other. The polysaccharide coat essentially adheres the extracellular collagen matrix to the phospholipid bilayer of the sarcolemma (Guyton & Hall 2006; Nelson & Cox 2008). The intercalated disks which bridge the extracellular space to create a continuous intracellular space are discrete structures found at intervals throughout the tissue; movement of the intracellular space will move the intercalated disks which will translate into a displacing force on the extracellular space (Guyton & Hall 2006; Nelson & Cox 2008). Membrane structures, such as ion channels and ligands, extend from the membrane into the extracellular space (Nelson & Cox 2008). Intracellular structures, such as the cytoskeleton and microtubules, may cross the membrane and extend into the extracellular space (Guyton & Hall 2006; Nelson & Cox 2008). Thus, intracellular displacements are mechanically translated into strain in the extracellular space. Similarly, these structures provide a route for which extracellular displacements are translated into strain in the intracellular space. I choose to model this coupling with a linear spring, characterized by the stiffness constant *K* (Puwal & Roth 2010; Punal & Roth 2012). The coupling force may be expanded in Taylor series; a linear spring constant is merely the lowest order term in the expansion. Taking advantage of the symmetry presented in the problem, the equations of mechanical equilibrium simplify to1516

These equations assert that non-zero relative displacements in the intracellular and the extracellular spaces will lead to a restoring force, the magnitude of which is proportional to the magnitude of the relative displacement (*u–w*) with the spring constant as that constant of proportionality. Consistent with Newton’s third law of motion, the force experienced by the extracellular space due to the intracellular space is equal in magnitude and opposite in sign to the force experienced by the intracellular space due to the extracellular space (Arya 1998), leading to the minus sign in the extracellular equation of mechanical equilibrium. Equations 15 and 16, along with Equations 7, 8, 10 and 11, are the coupled differential equations which we solve (subject to the appropriate boundary conditions) in order to obtain displacement fields ***u*** and ***w*** the hydrostatic pressure fields *p* and *q*.

### Incompressibility

Tissue and blood are mostly water (Guyton & Hall 2006; Nelson & Cox 2008), a fluid which is very nearly incompressible. So it is reasonable to include the condition of incompressibility on the displacement fields. The issue to consider is whether the intracellular and extracellular spaces are separately incompressible, wherein the intracellular (***u***) and extracellular (***w***) displacement fields are divergenceless.1718

or whether the spaces together exhibit incompressibility, wherein19

If the spaces together exhibit incompressibility, then volume changes in the intracellular space are accompanied by complementary volume changes in the extracellular space. When we consider that the elastic properties of the extracellular space arise from a strong covalently bonded lattice of collagen, the elastic deformation is likely not accompanied by volume changes of the extracellular space. We, therefore, make the *ad hoc* assumption20

Thus, we consider the intracellular and extracellular spaces separately incompressible (Eqs. 17 and 18). Taking advantage of the symmetry of the problem, and expressing the divergence in spherical coordinates2122

and so the displacements *u* and *w* are proportional to 1/r^2^.

### The cardiac cycle, time dependence of parameters, and quasi-static equilibrium

The contraction (systole) and relaxation (diastole) of the chambers of the heart are coordinated with each beat in a sequence of events that constitute the cardiac cycle (Guyton & Hall 2006). The stresses which the intracellular and extracellular spaces undergo vary over the course of the cardiac cycle. For example, the active tension is less in magnitude in cardiac tissue during the diastolic phase of the cardiac cycle than it is during the systolic phase. Therefore, the stresses, strains, active tension, and hydrostatic pressures are all functions of time. Chadwick (Chadwick 1982; Chadwick 1981) suggests *a priori* that the tissue is in quasi-static equilibrium at each instant of time. The validity of this assumption is supported by two observations. First, the elastic moduli are large in magnitude and displacement waves travel at relatively high speeds in media with large elastic moduli (Latimer et al. 2003; Lowrie 2007). Second, *post hoc* results predicated on the assumption of quasi-static equilibrium predict that displacements and hydrostatic pressures are very small in magnitude; perturbations of these quantities over the course of the cardiac cycle are even smaller. Small changes in displacements and hydrostatic pressures will, therefore, occur at a very rapid rate, essentially achieving quasi-static equilibrium.

We are interested in the average response of the heart tissue over the course of the cardiac cycle. We will, therefore, concern ourselves with the time average of the active tension *T*_0_ = <*T*(*t*)>, the time average of the chamber pressure *q*_*0*_ = <*q*_*c*_(*t*)>, and the time average of the pericardial pressure *q*_1_ = <*q*_*p*_(t)>. Elastic moduli and the coupling spring constant are assumed to be time-independent parameters.

### Geometry of the left ventricle

We will focus on using the left ventricle as our prototype for the single chamber, spherical heart since it is the largest chamber of the heart, in terms of both muscle mass and chamber volume (Chadwick 1982; Guyton & Hall 2006). Observations which allow us to characterize the dimensions of our spherical heart are as follows: As ventricular systole is initiated the atrioventricular valves close. The pressure in the left ventricle rises with increasing contraction until the pressure exceeds the diastolic pressure of the aorta at 80 mmHg. The semilunar valves open and blood is ejected. The left ventricular pressure may rise to 120 mmHg at peak systole. The volume of ejected blood is 70–90 mL, with 50 mL remaining in the left ventricle for a total volume of 120–140 mL (Guyton & Hall 2006). The normal adult has a left ventricle muscle wall thickness of approximately 1.1 cm (Guyton & Hall 2006; Peskin 1975). Lastly, encasing the heart is the fluid-filled pericardial sac (Guyton & Hall 2006; Morgan et al. 1965); pressures in the pericardium are on the order of a few millimeters of mercury (Morgan et al. 1965). For our single chamber, spherical heart we will select have an inner radius *a* = 3.14 *cm*, outer radius *b* = 4.24 *cm*, a time averaged outside (pericardial) pressure *q*_1_ = 2 mmHg, and a time averaged inside (chamber) pressure *q*_0_ = 100 mmHg (see Figure 2). A summary of parameters used is given in Table 1.

**Table 1:** **Table of parameters**

***Parameter***	***Value***	***Comment***
Chamber Pressure	*q*_*0*_ = 100 *mmHg*	A time-average value on the order of the chamber pressure during ventricular systole
Pericardial Pressure	*q*_*1*_*=* 2 *mmHg*	
Extracellular Shear Modulus	*G* = 10^4^*Pa*	
Ratio of Elastic Moduli	*μ/G* = 3	Based on Ohayan, et al. Implies *Δ* = Π in Eqs. 27 & 28
Active Tension	*T*_0_ = 100 *N/m*^2^	A time-average value on the order of systole for isolated myocytes (Chen & Varghese 2010)
Radius of Inner Chamber	*a* = 3.14 *cm*	Implies an inner chamber volume similar to the human left ventricle
Radius of Spherical Heart	*b* = 4.24 *cm*	Implies a wall thickness similar to the human left ventricle
Estimate of Coupling Parameter	*K***≳** 10^9^ − 10^10^*N*/*m*^3^	Based on experimental data of 5 to 6 *mm* displacement in canines, Eqs. 29 & 31, and Figure 3

**Figure 3:**
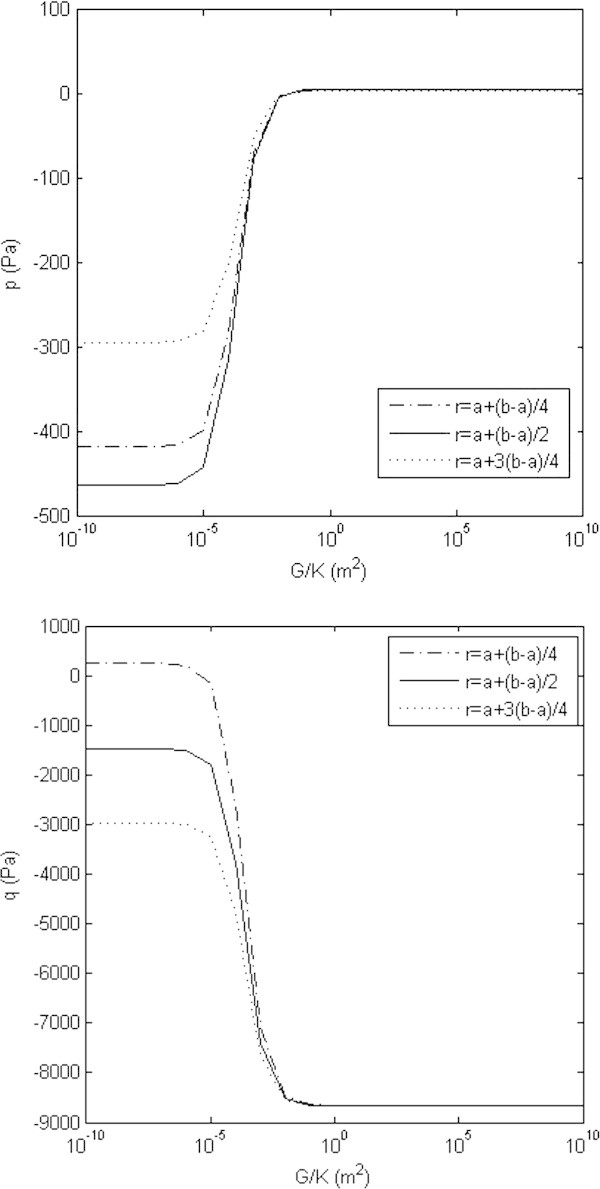
**Intracellular and extracellular displacement functions.** The product of the displacement functions *u* and *w* with the extracellular shear modulus G and the square of the radial position coordinate *r* are plotted as a function of the extracellular shear modulus *G* divided by the spring coupling constant *K*. Active tension is *T*_0_ = 100 *N/m*^2^ is maintained (Chen & Varghese 2010) and the ratio of the intracellular Young’s modulus *μ* to the extracellular shear modulus is given as *μ/G* = 3. Experimental data from canine hearts (Linke et al. 1994) indicates displacements on the order of a few millimeters; taken with the analysis presented here, this would suggest a very large value of *K*, on the order of 10^9^ or 10^10^*N/m*^2^.

**Figure 4:**
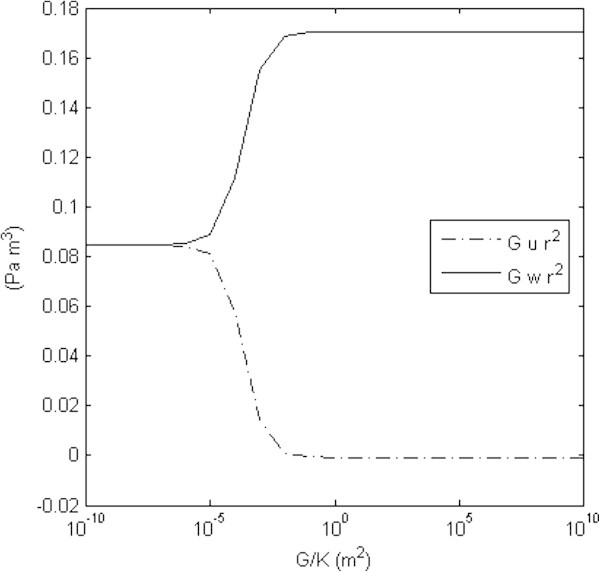
**The tissue hydrostatic pressures functions.** The intracellular (top plot) and extracellular (bottom plot) pressure functions are plotted as a function of the extracellular shear modulus *G* divided by the spring coupling constant *K*. Active tension is *T*_0_ = 100 *N/m*^2^ is maintained (Chen & Varghese 2010) and the ratio of the intracellular Young’s modulus *μ* to the extracellular shear modulus is given as *μ*/*G* = 3. The pressure curves follow a general relation (A + BG/K)/(C + DG/K), where *A, B, C,* and *D* and are constants; as shown, this relation holds for various depths within the heart wall. At the boundaries, intracellular pressure is zero and extracellular pressure is equal to the chamber and pericardial pressures for all values of *K*.

Values of chamber pressure, pericardial pressure, and active tension used are approximately time averaged over one cardiac cycle in order to discuss general trends in pressure and displacement relationships. Figures 3 and 4 assume the ratio of the intracellular Young’s modulus *μ* to the extracellular shear modulus *G* is approximately 3; while no particular value of extracellular shear modulus is assumed in these figures, a commonly accepted value is included here for reference. Analysis based on Figure 3 provides an estimate of the coupling spring constant *K*.

### Permanent deformations

We have assumed that we are in the region of reversible elasticity—that is, when the deforming stress is removed the elastic deformations return to zero. This is not always the case. The extracellular collagen mesh is formed from cross-linking covalent bonds. Each individual covalent bond, as we have said, may be approximated by a spring and the elastic continuum approach is based on a single preferred bond point. Under increasing stresses the cross-linking bonds may be broken, new bonds re-formed, and the deforming stress plastically deforms the collagen mesh. Active tension, pericardial pressure, and chamber pressure are not generally of sufficient magnitude to cause plastic deformation (Guyton & Hall 2006; Peskin 1975; Morgan et al. 1965).

## Results

We solve Equations 15 and 16 for our spherical heart shown in Figure 2 under the following boundary conditions on stress: The inner radius is *a* and the outer radius is *b*; the muscle wall is in the region *a < r < b*. The pericardial sac is represented by the region *r > b* and an average hydrostatic pressure *q*_1_ is maintained over the course of the cardiac cycle. In the heart chamber *r < a* an average hydrostatic pressure *q*_0_ is maintained. (We note that *q*_0_ > *q*_1_) The fluid in the spaces *r < a* and *r > b* are assumed to be continuous with the extracellular space, while there is, of course, no intracellular space in these regions. To find the displacement fields and hydrostatic pressures associated with these boundary pressures, we seek an analytical solution to Equations 15 and 16 that is consistent with the boundary conditions2324

These boundary conditions on stress describe an intracellular space that has free boundaries (Reismann & Pawlik 1980), capable of moving into the pericardial sac and the heart chamber. However, the extracellular space is subject to the conditions of fluid pressure at the boundaries (Reismann & Pawlik 1980). We will find it convenient to introduce several shorthand variables for use in our solution of the displacement fields and hydrostatic pressures:25262728

The intracellular displacement field and hydrostatic fluid pressure solve as2930

The extracellular displacement field and hydrostatic fluid pressure solve as3132

Displacements may be plotted (Figures 3 and 4) as a function of the spring constant. Experimentalists have noted displacements on the order of a few millimeters (Linke et al. 1994), consistent with a large value of K (on the order of 10^10^*N/m*^3^). Within the heart wall, a plot of pressures (Figure 4) versus *G/K* obeys a general relation (*A* + *BG/K*)/(*C* + *DG/K*), where *A, B, C,* and *D* are constants. It should be noted, with positive elastic moduli that  is less than zero as well, since *a < b*.

We find that coupling between the intracellular and extracellular spaces means that there is a minimum active tension which must be applied in order to cause displacement of the intracellular space. An active tension in the tissue nearly half the pressure difference between the chamber and the pericardium, offset by a factor from the geometry of the problem  and a factor from the elastic coupling Δ, causes zero intracellular displacement (*u* = 0) of the intracellular space.33

Larger active tensions are necessary to cause displacement of the intracellular spaces of the cardiomyocytes. While the intracellular space experiences no displacement under the active tension of Equation 33, the extracellular collagen matrix does, with a magnitude34

Expansion of the extracellular matrix under a specific active tension is not unexpected. The intracellular space was solved as a free boundary, while the extracellular space was solved with a stress continuous with the chamber and pericardial sac and where the pressure difference between them exerted an outward force. We also note that the intracellular space is not completely unperturbed by the active tension of Equation 33. In lieu of displacements, there is a buildup of hydrostatic pressure in the intracellular space35

There is a bound on the magnitude of the displacement and active tension for which the solutions of Equations 29, 30, 31 and 32 are valid. Consider an increasing active tension for a fixed chamber and pericardial pressure. As the active tension is increased, the intracellular displacement increases inward toward the origin. The chamber itself will collapse to zero volume if the displacement of the inner wall *u* (*r* = *a*) = –*a*. (Similarly, the extracellular space will collapse toward the origin with increasing active tension, though as we saw above there is an outward force, caused by the higher chamber pressure, that is greater in the extracellular space than in the intracellular space.) The upper bound on tension, therefore, is found when *u* (*a*) = –*a*. (But, of course, systemic physiological responses will resist the chamber collapsing, so the region of validity for these equations is narrower than this.)36

Further increases in active tension are accompanied by increases in hydrostatic pressure and cannot be accompanied by continued inward collapse; however, the exact functional form of the hydrostatic pressures will differ from that given by Equations 30 and 32.

### Hypertension

Hypertension is the most common cardiovascular condition, affecting an estimated 1 billion people worldwide. Among the symptoms the patient presents is an elevated blood pressure, typically greater than 140 mmHg systolic over 90 mmHg diastolic (Libby et al. 2008). Hypertrophy, or enlargement of the heart chamber, typically presents later in the course of the illness. In the large arteries of systemic circulation, this hypertrophy is accompanied by an increase in the size of the vascular smooth muscle cells and the accumulation of additional extracellular matrix proteins like collagen (Guyton & Hall 2006; Libby et al. 2008). Thus, the tissue not only elastically displaces under the action of increased blood pressure but also adapts through a remodeling of the muscle cells and extracellular matrix. Similar remodeling under hypertension induced hypertrophy has been observed in the chambers of the heart. Under such remodeling, parameters such as active tension and elastic moduli will be a function of time *t*.373839

where *τ* is the characteristic time for the remodeling mechanisms.

As a simplified model of elastic deformation under hypertension we shall assume the deformation is early in the course of the condition (such that *t* ≪ *τ*) and, therefore, hypertension is considered to simply be an increase in *q*_0_. (Since the pericardial sac is sufficiently isolated from systemic circulation, the pericardial pressure negligibly increases with the increased blood pressure (Guyton & Hall 2006; Morgan et al. 1965; Libby et al. 2008). Observe the displacement fields and the hydrostatic pressures are linearly proportional to this chamber pressure *q*_0_ so that, for all *r* in the range *a < r < b* there is an increase in the intracellular and extracellular displacements and hydrostatic pressures that is proportional to the increase of the chamber pressure. Thus, early in the course of illness for a hypertensive patient there is an outward directed force due to the high chamber pressure; later compensatory hypertrophy may then be considered as being in direct response to this outward directed force.

A bidomain approach allows us, in our description of strain, to separately consider the collagen matrix and the intracellular actin-myosin complex. With a mathematical description of the strains in both spaces we may subsequently consider, for example, whether the process of compensatory hypertrophy is centrally concerned with the intracellular space and remodeling of the extracellular collagen matrix is incidental (i.e. that there is a single process at work) or whether there are two compensatory processes that respond to strain separately, but simultaneously in each space.

### Hypovolemic hypotension

Hypovolemia, or decreased blood volume, is almost always accompanied by hypotension, or decreased blood pressure. Hypovolemic hypotension will present with decreased chamber pressure (Guyton & Hall 2006; Libby et al. 2008). Thus, the hypotensive elastic response predicted is for a smaller outward directed force on the extracellular collagen matrix and functionally similar displacement and pressure fields. Equations 29 and 31 describe the displacement fields; observe that with a sufficiently small chamber pressure, an increased inward contraction of the muscle wall will result. With the same pericardial pressures and active tensions, the inward contraction of the muscle wall will be of greater magnitude than that experienced by the non-hyoptensive patient.

### Hypertrophic cardiomyopathy

Hypertrophic Cardiomyopathy (HCM) is the most common genetically transmitted cardiovascular disease. HCM presents with a constellation of symptoms, including sudden cardiac death. In HCM there is a non-dilational thickening of the wall of the left ventricle (LV). Postmortem assessment shows LV wall thickness nearly doubles, though the thickening is often asymmetrical (Guyton & Hall 2006; Libby et al. 2008). A non-dilational thickening of the wall may be approximated as symmetrical and taken as in increase in the outer wall radius *b* in Equations 29, 30, 31 and 32. Observe in the displacement fields that the factor *ln(b)* is multiplied by the active tension *T*_0_; we conclude that smaller active tensions are predicted to be necessary in HCM hearts to cause similar deformations as non-HCM hearts. In other words, it is predicted that a more massive muscle can cause the same deformation with less required tension. Meanwhile, in the hydrostatic pressures the geometric shorthand variables *l*^2^ and *z* are multiplied by (*q*_*0*_ – *q*_*1*_); larger pressure differences between the chamber and pericardium are, thus, predicted to be necessary in HCM hearts to cause the same hydrostatic pressures within the tissue as non-HCM hearts. In other words, it is necessary to put a bigger pressure differential across a more massive heart to achieve the same distribution of pressure within. Note that these predictions presume the elastic parameters of HCM and non-HCM hearts are identical. This may not be a valid assumption after the tissue is remodeled.

### Dilated cardiomyopathy

Dilated Cardiomyopathy (DCM) is the most common of all cardiomyopathies. DCM presents with enlargement and impaired contraction of one or both of the ventricles. Its origin may be genetic, immunological, chemical, or otherwise idiopathic. As the chamber dilates, there is compensatory thickening of the ventricular walls to maintain cardiac output (Guyton & Hall 2006; Libby et al. 2008). If we consider DCM early in its course, before compensatory thickening has taken place, then the volume of the muscle wall will remain the same. That is, the inner radius of the chamber and the outer radius of the chamber will vary in such a way that the volume between them remains constant. Specifically, if the initial inner radius is *a*_0_, the initial outer radius is *b*_0_, and the dilated inner radius is *a*_1_, then the dilated outer radius will be *b*_1_ where40

Quantities such as , *z*, and *l*^2^ increase with increasing *a*_1_. Equations 29, 30, 31 and 32 predict that similar displacements will occur for smaller active tension *T*_0_ than in a non-DCM heart. However, again note that these predictions presume the elastic parameters of DCM and non-DCM hearts are identical. This may not be a valid assumption after the tissue has remodeled.

## Conclusion

The two-domain approach to elastic deformation of cardiac muscle allows us to separately analyze displacements and hydrostatic pressures of the intracellular and extracellular spaces. We considered the deformations and pressures applied to four important pathologies: hypertension, hypotension, HCM, and DCM. The human heart is not a spherical, single chamber heart. Here, the geometry, elastic properties of the muscle, and deforming stresses of the pathology exhibit angular and azimuthal symmetry; this simplification is intended to facilitate the determination of an analytic solution that describes the effects of radially directed pressures and tangential active tension in the muscle may be analyzed. In reality, the largest chamber of the human heart is the left ventricle, which is approximately conical in shape and resembling a folded sheet of cardiac tissue. Intercalated disks bridge the cells in the radial direction as well as the tangential direction, providing background isotropy of the intracellular space and a radially directed active tension. Atria empty into the ventricles and the ventricles, in turn, empty into arteries; thus, the stresses are not uniform over the outer surface of the heart (pericardial sac). The great stresses around the aorta, for example, are not considered here.

In spite of these limitations, the results presented here are generally consistent with our expectations. In the hypertensive example, for instance, the deforming stress is a relative net outward force (greater chamber pressure, unchanged pericardial pressure) on the extracellular collagen mesh; the spring coupling between the two continua translates this outward force to the intracellular muscle. It is not surprising, therefore, that the extracellular collagen mesh deforms to a greater extent than the intracellular space. I propose that one method of measuring the coupling spring constant is to image the microstructure of pre- and early post-hypertensive cardiac chambers. By determining the relative extent of deformation of the intracellular and extracellular spaces Eqs. 29 and 31 can be employed to provide an approximate magnitude of *K*.

Under long term deforming stress (e.g. untreated hypertension) there is a sustained hydrostatic pressure differential across the membrane (Eqs. 30 and 32 are related to the true pressures by the volume fraction of the two spaces). Given long term pressure differences, there is the potential for bulk fluid flow between the two spaces. This can potentially be a mechanism by which the tissue remodels itself (i.e. how the magnitudes of the elastic moduli change).

One significant future application of this model is likely to require numerical evaluation: It is known that a two-domain treatment of the electrical behavior of heart muscle may exhibit pro-arrhythmic behavior at a distance with electrical stimulation. There is the potential for mechanical perturbations (e.g. multiple ischemic regions distributed through the muscle) to translate their deforming stresses along the collagen matrix and combine in a non-ischemic region, thereby activating stretch-activated membrane channels. The activation of these stretch-activated channels in an otherwise healthy region of tissue will lead to ectopic beats and, if activated in a quasi-refractory state, may trigger rotary wave activity (i.e. fibrillation). Thus, the chief benefit of a two-domain elastic model is to permit us to consider the mechanical behavior of the muscle averaged over many cells (the tissue approach) while still separately considering the separate spaces (the cellular approach). It is with such a model that we are able to consider whole tissue behavior and predict cellular-level dynamics.
